# Long non-coding RNA PVT1 knockdown suppresses fibroblast-like synoviocyte inflammation and induces apoptosis in rheumatoid arthritis through demethylation of *sirt6*

**DOI:** 10.1186/s13036-019-0184-1

**Published:** 2019-07-02

**Authors:** Chun-Wang Zhang, Xia Wu, Dan Liu, Wei Zhou, Wei Tan, Yu-Xuan Fang, Yu Zhang, Yan-Qing Liu, Guo-Qing Li

**Affiliations:** 1 Department of Rheumatology, Affiliated Hospital of Yangzhou University, No. 368, Hangjiang Road, Yangzhou, 225000 Jiangsu Province People’s Republic of China; 2 Clinical Medical College, Dalian Medical University, Dalian, 116044 People’s Republic of China; 3 Department of Pathology, Clinical Medical College, Yangzhou University, Yangzhou, 225000 People’s Republic of China; 4 Medical College of Yangzhou University, Yangzhou, 225000 People’s Republic of China

**Keywords:** Long non-coding RNA plasmacytoma variant translocation 1, Sirtuin 6, Rheumatoid arthritis, Methylation, Inflammation, Apoptosis

## Abstract

**Background:**

As a type of chronic autoimmune joint disease, rheumatoid arthritis (RA) is a disorder, characterized by a variety of physical symptoms as well as RA fibroblast-like synoviocyte (RA-FLS) proliferation. More recently, long non-coding RNAs (lncRNAs) have been implicated in the progression of various diseases including the progression of RA. Hence, the aim of the current study was to investigate the role by which the lncRNA, plasmacytoma variant translocation 1 (PVT1), influences RA-FLSs and its ability to modulate the methylation of *sirtuin 6 (sirt6)*.

**Methods:**

RA rat models were initially established to determine the expression of PVT1 and *sirt6* in synovial tissues and RA-FLSs. Elevation or depletion of PVT1 or *sirt6* was achieved by means of transformation with plasmids in order to investigate their effects on RA-FLS proliferation, inflammation and apoptosis. The localization of PVT1 and its binding ability to the *sirt6* promoter region were also explored in an attempt to elucidate the correlation between PVT1 and *sirt6* methylation.

**Results:**

High expression of PVT1 and low expression of *sirt6* were detected in the synovial tissues and RA-FLSs of the rat models. RA-FLSs treated with sh-PVT1 or oe-sirt6 exhibited suppressed cell proliferation, inflammation and induced apoptosis. PVT1 was predominately localized in the nucleus while evidence was obtained indicating that it could bind to the *sirt6* promoter to induce *sirt6* methylation, thus inhibiting *sirt6* transcription. PVT1 knockdown was observed to restore *sirt6* expression through decreasing *sirt6* methylation, thereby alleviating RA.

**Conclusion:**

The key findings of the study provide evidence suggesting that, PVT1 knockdown is able to restrain RA progression by inhibiting *sirt6* methylation to restore its expression.

## Background

Rheumatoid arthritis (RA) represents a type of autoimmune joint inflammatory disorder and has been reported to affect approximately 6% of people over 65 years old, while showing predominance in women more so than men [1]. The chief characteristics of RA include hyperplasia synovitis and inflammatory immune cell infiltration [2]. The disease is often accompanied by invasiveness of macrophages, synovial fibroblasts (SFs), T lymphocytes, and B lymphocytes/plasma cells, which consequently results in the perpetuation of joint destruction in RA, which if left untreated, often leads to disability, decrease in quality of life as well as elevated comorbidity [3]. RA fibroblast-like synoviocytes (RA-FLSs) are predominant component that have been implicated in the initiation, progression and perpetuation of joint destruction inflammation. Moreover, activation of RA-FLS phenotype is reflected by the pro-inflammatory milieu, with studies earmarking RA-FLS as dominant promoter of inflammation [4]. Currently, therapies that target predominant cytokines and RA-dependent inflammatory mediators can temporarily relief pain [5]. Accordingly, improved knowledge of inflammation in RA will help to understand disease pathogenesis, which can be eventually used for RA treatments and/or preventions.

Studies have highlighted the involvement of long non-coding RNAs (lncRNAs) in an array of biologic processes, emphasizing their dysregulation with disease elements such as cancer cell invasion, proliferation and metastasis [6]. LncRNA plasmacytoma variant translocation 1 (PVT1) is suggested to promote tumorigenesis in several cancers including ovarian cancer, colorectal cancer, and lung cancer, among which PVT1 is upregulated and induced cell proliferation and invasion [7–9]. LncRNA may function as a regulator in inflammatory responses in physiologic and pathologic processes [10]. Differential expression of lncRNAs have been analyzed in RA tissues and cells, with evidence obtained indicating that the GAPLINC is up-regulated in RA-FLS [11] with the downregulation of HOX transcript antisense intergenic RNA in LPS-induced chondrocytes [12]. Most importantly, previous literature has highlighted that the upregulation of PVT1 promotes chondrocyte apoptosis in osteoarthritis [13], emphasizing the potential role of PVT1 in RA. *Sirtuin 6* (*sirt6*) has been confirmed to be a target of PVT1, whereby PVT1 binds to the *sirt6* promoter region. Sirts are regulators of various cellular and molecular processes including cell survival, gene transcription, and inflammation [14]. Moreover, the overexpression of *sirt6* has been reported to suppress the inflammatory response as well as bone destruction of collagen-induced arthritis model in mice [15]. Hypermethylation of *sirt6* has been demonstrated in ductal carcinoma in situ [16]. Based on the aforementioned exploration of literature, we hypothesized that PVT1 may affect the biological functions of RA-FLSs via regulation of the methylation of *sirt6* in its promoter region. Hence, the aim of the current study was to investigate the effect of PVT1 on the proliferation, inflammation and apoptosis in RA-FLS of RA in order to elucidate the molecular mechanisms associated with RA.

## Results

### Successful establishment of RA rat model

**Fig. 1:**
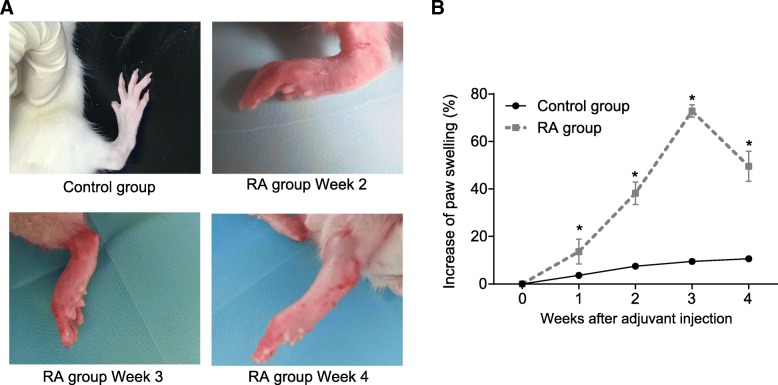
RA model in rats was successfully established. **a**, observation of rat hind paw; **b**, a chart of increase of paw swelling (%); ^*^p < 0.05 vs. the control group; the control group = 10, the RA group = 12. All data were measurement data and presented as mean ± standard derivation; the data comparison at different time points was processed with repeated measures analysis of variance. RA, rheumatoid arthritis

In order to examine the RA models, the physical characteristics of the RA rats as well as the rats in the control group were observed. The rats in the control group were noted to have a normal diet, good spirit, agile movement and strong limbs after miner oil injection, with no clinical symptoms detected during the 3rd week after injection (Fig. 1a). In the RA group, 12 rats exhibited several clinical symptoms including swelling and redness of hind paw on the 10th day after CFA injection, which were found to be more severe by the 2nd week, before subsiding after the 3rd week and complete alleviation by the 4th week. No swelling or redness around the hind paw of the other 3 rats in the RA group was identified. Therefore, we concluded that the success rate of RA rat model was 80%. In addition, the results of the increase of paw swelling (%) of rats in the RA group compared with the 0th day were shown in Fig. 1b. All these results suggested the RA model in rats was successfully established.

### PVT1 is highly expressed and *sirt6* is poorly expressed in synovial tissues of RA rats

**Fig. 2:**
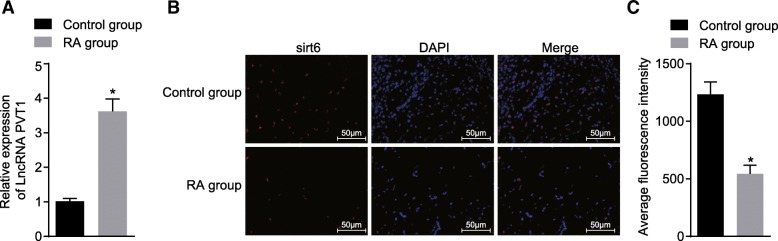
High expression of PVT1 and low expression of *sirt6* in synovial tissues of RA rats. **a**, the PVT1 expression in synovial tissues of rats in the control and RA groups determined by RT-qPCR; ^*^p < 0.05 vs. the control group; **b**, the *sirt6* expression in synovial tissues of rats in the control and RA groups quantified by immunofluorescence (× 200); **c**, a bar chart showing the *sirt6* expression in synovial tissue of rats in the control and RA groups; ^*^p < 0.05 vs. the control group; the control group = 10, the RA group = 12. The experiment was repeated 3 times to obtain the mean values; the gene expression levels and the fluorescence values were presented as mean ± standard derivation; independent *t*-test was performed for comparison between two groups. PVT1, long non-coding RNA plasmacytoma variant translocation 1; Sirt6, sirtuin 6; RA, rheumatoid arthritis; RT-qPCR, reverse transcription quantitative polymerase chain reaction

Next, the synovial tissues were extracted from rats in the control and RA groups in order to determine the expression of PVT1 and *sirt6* by means of RT-qPCR (Fig. 2a) and Immunohistochemistry (Fig. 2b, c), respectively. The results revealed the RA group had a significantly higher PVT1 expression than the control group (*p* < 0.05). The nucleus was blue at ultraviolet excitation after counterstaining with DAPI, while the positive protein expression was red. The RA group had a markedly lower *sirt6* expression when compared to the control group (*p* < 0.05). The results obtained suggested that the high expression of PVT1 and low expression of *sirt6* in the synovial tissues of RA rats could be associated with the development and progression of RA.

### Successful isolation of RA-FLSs

**Fig. 3:**
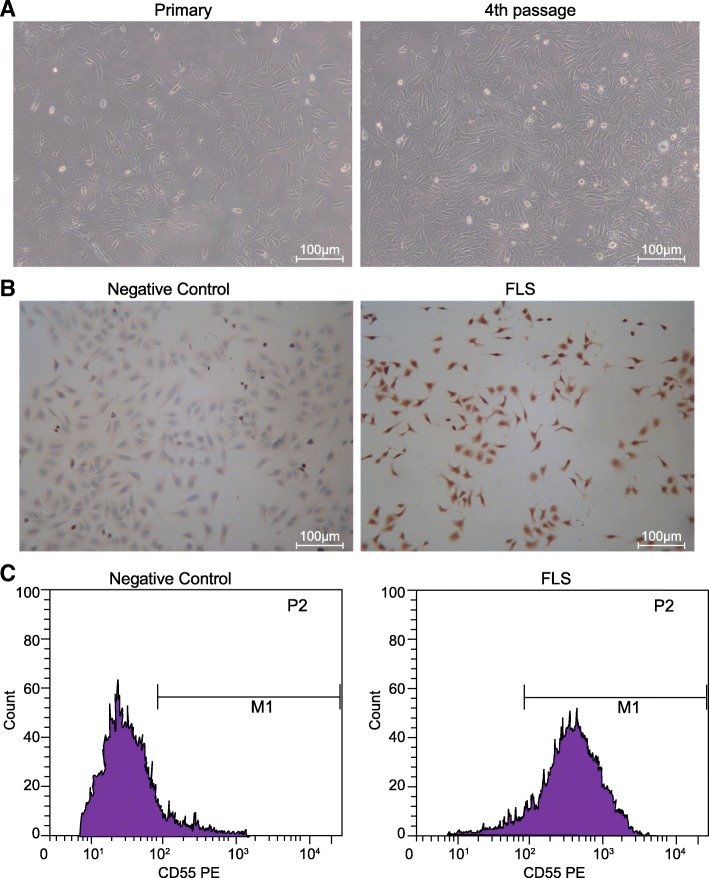
RA-FLSs in vitro culture and verification. **a**, the morphological changes of RA-FLSs in the primary and 4th passage observed under the inverted microscope; **b**, the Vimentin expression in RA-FLSs in the 4th passage determined by Immunohistochemistry; **c**, the percentage of CD55^+^ RA-FLSs in the 4th passage measured by flow cytometry. RA-FLSs, rheumatoid arthritis fibroblast-like synoviocytes

In order to ascertain as to whether the isolated cells were RA-FLSs, the RA-FLS surface biomarker CD55 and Vimentin were detected through the application of flow cytometry and Immunohistochemistry, respectively. The morphological changes of RA-FLSs in the primary and 4th passage were observed under an inverted microscope: the RA-FLSs had a triangle or diamond shape in which the nucleus was found to be orbicular-ovate like and located on the central of RA-FLSs with a clear nucleolus, and surrounded by secretion aggregation (Fig. 3a). The Vimentin immunohistochemistry results (Fig. 3b) suggested that the staining results of RA-FLSs in the 3rd and 4th passage were positive, with a significant degree of brownish yellow granules detected in the cytoplasm. The flow cytometry results (Fig. 3c) revealed that the percentage of CD55^+^ RA-FLSs in the 3rd and 4th passage was (94.61 ± 3.67)%. The great majority of the isolated cells were confirmed to be RA-FLSs indicating by positive Vimentin and strong positive CD55 from the obtained results.

### PVT1 knockdown suppresses RA-FLS proliferation and inflammation yet promoting apoptosis

**Fig. 4:**
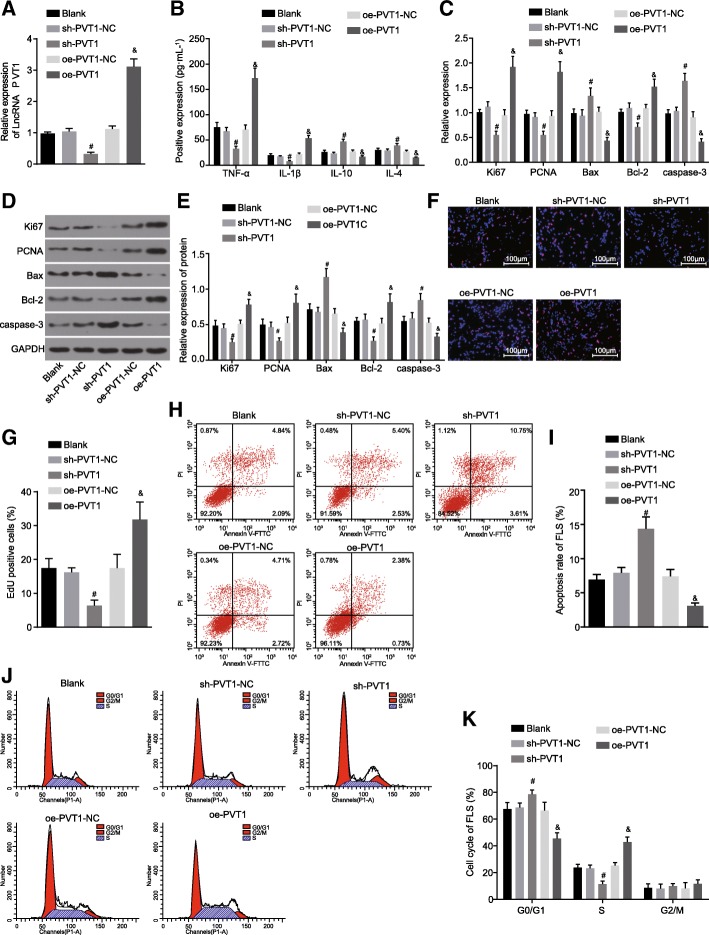
RA-FLS proliferation and inflammation were hindered and apoptosis was promoted by the knockdown of PVT1. **a**, the PVT1 expression in RA-FLSs determined by RT-qPCR; **b**, the serum levels of TNF-α, IL-1β, IL-10 and IL-4 measured by ELISA; **c**, the mRNA expression of Ki67, PCNA, Bcl-2, Bax and caspase-3 in RA-FLSs assessed by RT-qPCR; **d**, the protein levels of Ki67, PCNA, Bcl-2, Bax and caspase-3 assessed by western blot analysis; **e**, the protein levels of Ki67, PCNA, Bcl-2, Bax and caspase-3 in RA-FLSs; **f**, the RA-FLS proliferation determined by EDU staining (× 100); **g**, a bar chart of EDU positive stained RA-FLSs; **h**, the RA-FLS apoptosis evaluated by flow cytometry; **i**, a bar chart of apoptosis rate of RA-FLSs; **j**, the cell cycle distribution of RA-FLSs assessed by flow cytometry; **k**, a bar chart of cell cycle of RA-FLSs; ^#^ *p* < 0.05 vs. the sh-PVT1-NC group; ^&^ *p* < 0.05 vs. the oe-PVT1-NC group. The results of quantitative analysis were presented as mean ± standard derivation which compared by one-way analysis of variance; the experimental data was obtained from the average of 5 independent experiments. PVT1, long non-coding RNA plasmacytoma variant translocation 1; Sirt6, sirtuin 6; RA-FLSs, rheumatoid arthritis fibroblast-like synoviocytes; PCNA, proliferating cell nuclear antigen; Bcl-2, B-cell lymphoma/leukemia 2; Bax, Bcl-2-associated X protein; GAPDH, glyceraldehyde-3-phosphate dehydrogenase; TNF-α, tumor necrosis factor-α; IL-1β, interleukin-1β; IL-10, interleukin-10; IL-4, interleukin-4; EDU, 5-Ethynyl-2′-deoxyuridine; PI, propidium iodide; FITC, fluorescein isothiocyanate; ELISA, enzyme-linked immunosorbent assay; RT-qPCR, reverse transcription quantitative polymerase chain reaction; NC, negative control

In order to investigate the mechanism of PVT1 in RA-FLS inflammation, proliferation and apoptosis, the expression levels of TNF-α, IL-1β, IL-10, IL-4, Ki67, PCNA, Bcl-2, Bax and caspase-3 in the blank, sh-PVT1-NC, sh-PVT1, oe-PVT1-NC and oe-PVT1 groups were detected through the application of ELISA, RT-qPCR, western blot analysis, EDU staining and flow cytometry. The PVT1 expression in RA-FLSs post transfection was determined by RT-qPCR means, which indicated that the expression of PVT1 was lower in the sh-PVT1 group when compared with the sh-PVT1-NC group (*p* < 0.05), while higher levels were identified in the oe-PVT1 group in comparison with the oe-PVT1-NC group (*p* < 0.05) (Fig. 4a). The results obtained demonstrated that the PVT1 expression in RA-FLSs was notably influenced by sh-PVT1 or oe-PVT1. Compared with the oe-PVT1-NC group, the oe-PVT1 group displayed significantly elevated serum levels of TNF-α and IL-1β, along with decreased levels of IL-10 and IL-4 (Fig. 4b); distinct increases in the mRNA expression and protein levels of Ki67, PCNA and Bcl-2, along with diminished expressions of Bax and caspase-3 were identified (Fig. 4c-e); enhanced cell proliferation (Fig. 4f and g); inhibited cell apoptosis (Fig. 4h and i); decreased cell percentage at G0/G1 phase, along with increased cell percentage at S phase (Fig. 4j and k) (all *p* < 0.05). Our results revealed there to be a contrasting trend in the sh-PVT1 group when compared to that of the sh-PVT1-NC group (all *p* < 0.05), which presented with significantly decreased serum levels of TNF-α and IL-1β, along with increased levels of IL-10 and IL-4 (Fig. 4b); markedly decreased mRNA expression and protein levels of Ki67, PCNA and Bcl-2, along with elevated expression levels of Bax and caspase-3 (Fig. 4c-e); restrained cell proliferation (Fig. 4f and g); induced cell apoptosis (Fig. 4h and i); considerably increased percentage of cells at the G0/G1 phase while a decreased percentage of cells were detected at the S phase (Fig. 4j and k). Taken together, it was concluded based on the results that proliferation and inflammation of RA-FLSs were suppressed by PVT1 knockdown, which was accompanied by stimulated cell apoptosis.

### Overexpression of *sirt6* restrains RA-FLS proliferation and inflammation yet inducing apoptosis

**Fig. 5:**
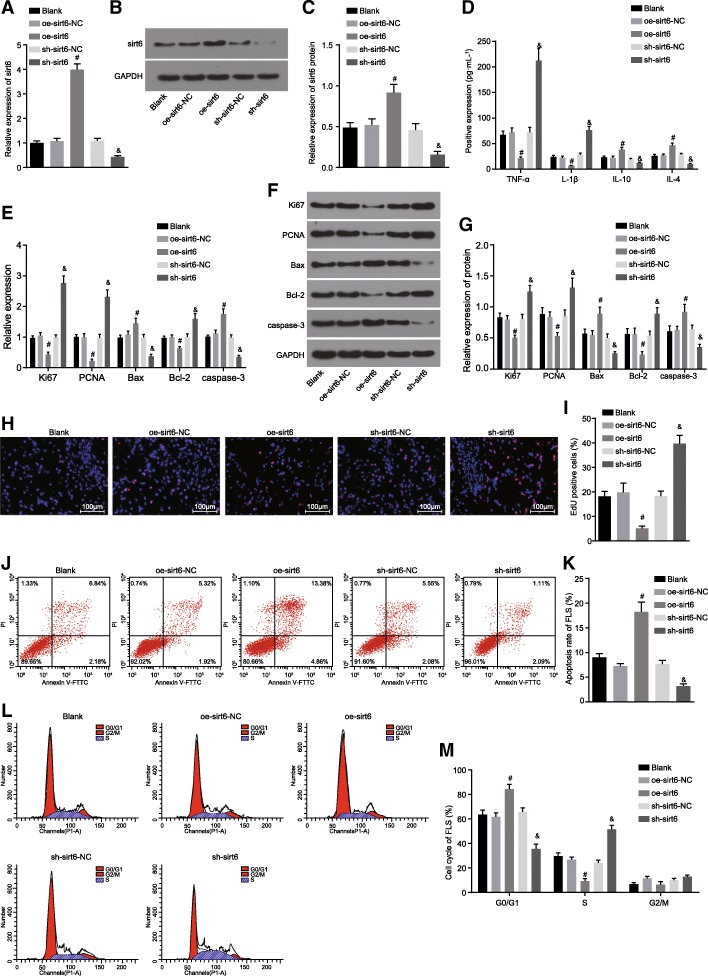
RA-FLS proliferation and inflammation were suppressed and apoptosis was promoted by overexpression of *sirt6* in RA. **a**, the mRNA expression of *sirt6* in RA-FLSs determined by RT-qPCR; **b**, the protein level of *sirt6* in RA-FLSs quantified by western blot analysis; **c**, the protein level of *sirt6* in RA-FLSs; **d**, the serum levels of TNF-α, IL-1β, IL-10 and IL-4 measured by ELISA; **e**, the mRNA expression of Ki67, PCNA, Bcl-2, Bax and caspase-3 in RA-FLSs evaluated by RT-qPCR; **f**, the protein levels of Ki67, PCNA, Bcl-2, Bax and caspase-3 assessed by western blot analysis; **g**, protein levels of Ki67, PCNA, Bcl-2, Bax and caspase-3 in RA-FLSs; **h**, the RA-FLS proliferation levels determined by EDU staining (× 100); **i**, EDU positive RA-FLSs assessed by EDU assay; **j**, the RA-FLS apoptosis evaluated by flow cytometry; **k**, a bar chart of apoptosis rate of RA-FLSs; **l**, the cell cycle distribution of RA-FLSs as assessed by flow cytometry; **m**, a bar chart of cell cycle of RA-FLSs; ^#^ *p* < 0.05 vs. the oe-sirt6-NC group; ^&^ *p* < 0.05 vs. the sh-sirt6-NC group. The results of quantitative analysis were presented as mean ± standard derivation; one-way analysis of variance was performed for comparison between the treatment group and the control group; the experimental data was obtained from the average of 5 independent experiments. Sirt6, sirtuin 6; RA-FLSs, rheumatoid arthritis fibroblast-like synoviocytes; PCNA, proliferating cell nuclear antigen; Bcl-2, B-cell lymphoma/leukemia 2; Bax, Bcl-2-associated X protein; GAPDH, glyceraldehyde-3-phosphate dehydrogenase; TNF-α, tumor necrosis factor-α; IL-1β, interleukin-1β; IL-10, interleukin-10; IL-4, interleukin-4; EDU, 5-Ethynyl-2′-deoxyuridine; PI, propidium iodide; FITC, fluorescein isothiocyanate; ELISA, enzyme-linked immunosorbent assay; RT-qPCR, reverse transcription quantitative polymerase chain reaction; NC, negative control

In order to further examine the mechanism by which *sirt6* influences RA-FLS inflammation, proliferation and apoptosis, the expression of TNF-α, IL-1β, IL-10, IL-4, Ki67, PCNA, Bcl-2, Bax and caspase-3 in the blank, oe-sirt6-NC, oe-sirt6, sh-sirt6-NC and sh-sirt6 groups was evaluated by ELISA, RT-qPCR, western blot analysis, EDU staining and flow cytometry. The *sirt6* expression in RA-FLSs after transfection (Fig. 5a-c) revealed that the mRNA expression and protein levels of *sirt6* were higher in the oe-sirt6 group than the oe-sirt6-NC group (*p* < 0.05), however the mRNA and protein levels were lower in the sh-sirt6 group when compared to the sh-sirt6-NC group (*p* < 0.05). These results suggested that the *sirt6* expression in RA-FLSs was significantly influenced by sh-sirt6 or oe-sirt6. Compared with the sh-sirt6-NC group, the sh-sirt6 group exhibited elevated serum levels of TNF-α and IL-1β and decreased levels of IL-10 and IL-4 (Fig. 5d); significantly elevated mRNA expression and protein levels of Ki67, PCNA and Bcl-2, while reduced levels of Bax and caspase-3 (Fig. 5c-g); promoted cell proliferation (Fig. 5h and i); suppressed cell apoptosis (Fig. 5j and k); notably decreased cell percentage at G0/G1 phase, and increased cell percentage at the S phase (Fig. 5l and m) (all *p* < 0.05). The serum levels of TNF-α and IL-1β were decreased, while the levels of IL-10 and IL-4 increased (Fig. 5d); mRNA expression and protein levels of Ki67, PCNA and Bcl-2 were decreased, while the levels of Bax and caspase-3 were elevated (Fig. 5c-g) in the oe-sirt6 group with significantly restrained cell proliferation (Fig. 5h and i), facilitated cell apoptosis (Fig. 5j and k) and an increased percentage of cells at the G0/G1 phase along with decreased cell percentage at S phase (Fig. 5l and m) in contrast to the oe-sirt6-NC group (all *p* < 0.05). The aforementioned results indicated that proliferation and inflammation of RA-FLSs were inhibited while cell apoptosis was induced in the event of *sirt6* overexpression.

### Enhanced RA-FLS proliferation and inflammation and suppressed apoptosis from PVT1 overexpression are attenuated by overexpressing *sirt6*

**Fig. 6:**
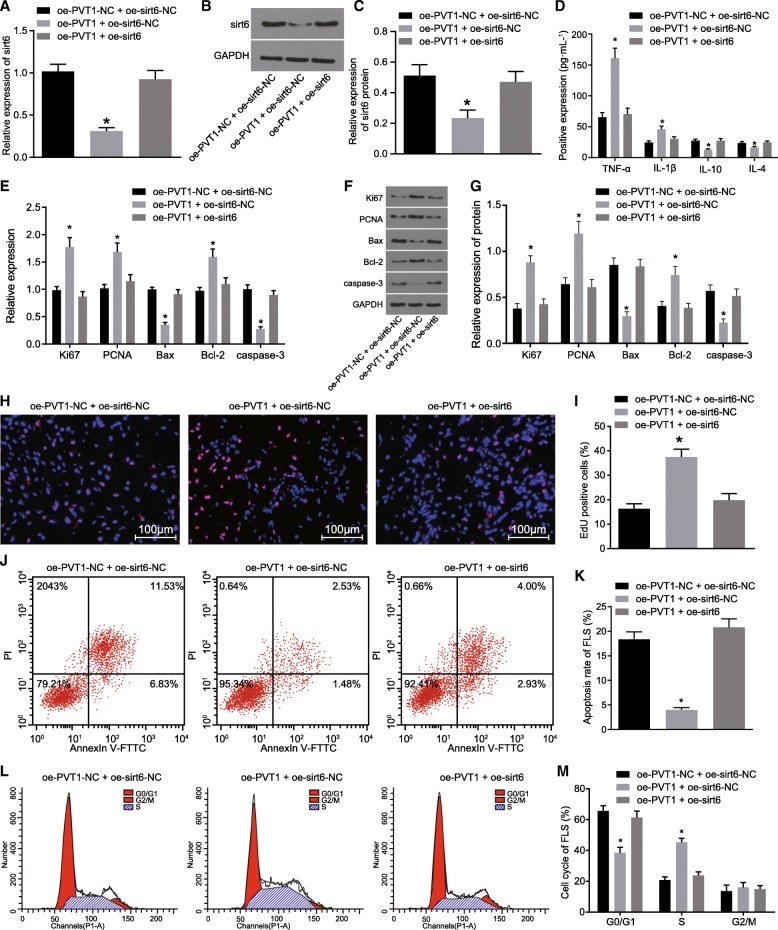
Overexpression of PVT1 promoted RA-FLS proliferation and inflammation and inhibited apoptosis, which was reversed by overexpression of *sirt6* in RA. **a**, the mRNA expression of *sirt6* in RA-FLSs determined by RT-qPCR; **b**, the protein level of *sirt6* in RA-FLSs measured by western blot analysis; **c**, the protein level of *sirt6* in RA-FLSs; **d**, the serum levels of TNF-α, IL-1β, IL-10 and IL-4 measured by ELISA; **e**, the mRNA expression of Ki67, PCNA, Bcl-2, Bax and caspase-3 in RA-FLSs assessed by RT-qPCR; **f**, the protein levels of Ki67, PCNA, Bcl-2, Bax and caspase-3 in RA-FLSs assessed by western blot analysis; **g**, the protein levels of Ki67, PCNA, Bcl-2, Bax and caspase-3 in RA-FLSs; **h**, the RA-FLS proliferation determined by EDU staining (× 100); **i**, a bar chart of EDU positive RA-FLSs; **j,** the RA-FLS apoptosis evaluated by flow cytometry; **k**, a bar chart of apoptosis rate of RA-FLSs; **l**, the cell cycle distribution of RA-FLSs assessed by flow cytometry; **m**, a bar chart of cell cycle of RA-FLSs; ^#^ *p* < 0.05 vs. the oe-sirt6-NC group; ^*^ *p* < 0.05 vs. the oe-PVT1-NC + oe-sirt6-NC group. The results of quantitative analysis were presented as mean ± standard derivation, which compared by one-way analysis of variance; the experimental data was obtained from the average of 3 independent experiments. PVT1, long non-coding RNA plasmacytoma variant translocation 1; Sirt6, sirtuin 6; RA-FLSs, rheumatoid arthritis fibroblast-like synoviocytes; PCNA, proliferating cell nuclear antigen; Bcl-2, B-cell lymphoma/leukemia 2; Bax, Bcl-2-associated X protein; GAPDH, glyceraldehyde-3-phosphate dehydrogenase; TNF-α, tumor necrosis factor-α; IL-1β, interleukin-1β; IL-10, interleukin-10; IL-4, interleukin-4; EDU, 5-Ethynyl-2′-deoxyuridine; PI, propidium iodide; FITC, fluorescein isothiocyanate; ELISA, enzyme-linked immunosorbent assay; RT-qPCR, reverse transcription quantitative polymerase chain reaction; NC, negative control

In order to examine the rescue effect associated with the overexpression of *sirt6* on the overexpression of PVT1 in RA-FLSs, the expression of TNF-α, IL-1β, IL-10, IL-4, Ki67, PCNA, Bcl-2, Bax and caspase-3 in the oe-PVT1-NC + oe-sirt6-NC, oe-PVT1 + oe-sirt6-NC and oe-PVT1 + oe-sirt6 groups was measured by ELISA, RT-qPCR, western blot analysis, EDU staining and flow cytometry to analyze the effect of PVT1 on RA-FLS proliferation, inflammation and apoptosis. The results of RT-qPCR (Fig. 6a) and western blot analysis (Fig. 6b, c) suggested that the mRNA expression and protein levels of *sirt6* were significantly depleted in the oe-PVT1 + oe-sirt6-NC group compared with the oe-PVT1-NC + oe-sirt6-NC group (*p* < 0.05); no significant difference was detected in relation to the mRNA expression and protein levels of *sirt6* in the oe-PVT1 + oe-sirt6 group (*p* > 0.05). In contrast to the oe-PVT1-NC + oe-sirt6-NC group, the oe-PVT1 + oe-sirt6-NC group exhibited a significantly increased serum levels of TNF-α and IL-1β, along with decreased serum levels of IL-10 and IL-4 (Fig. 6d); elevated mRNA expression and protein levels of Ki67, PCNA and Bcl-2, along with reduced levels of Bax and caspase-3 (Fig. 6c-g); promoted cell proliferation (Fig. 6h and i); suppressed cell apoptosis (Fig. 6j and k); decreased cell percentage at G0/G1 phase significantly, and an elevated percentage of cells at the S phase (Fig. 6l and m) (all *p* < 0.05). No significant difference was observed between the oe-PVT1 + oe-sirt6 and oe-PVT1-NC + oe-sirt6-NC groups (*p* > 0.05). The above results demonstrated that the promoted RA-FLS proliferation and inflammation and reduced apoptosis, as a result of overexpressed PVT1, which could be reversed by overexpressing *sirt6*.

### PVT1 inhibits *sirt6* expression by promoting *sirt6* promoter methylation

**Fig. 7:**
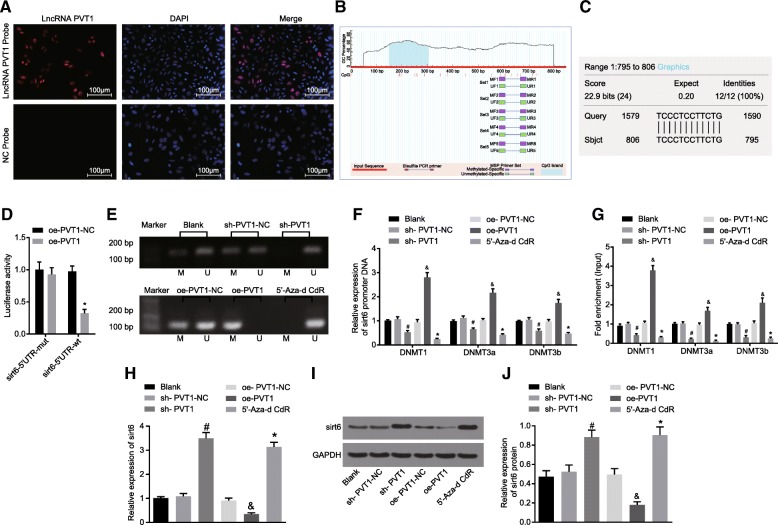
PVT1 inhibited *sirt6* expression by promoting *sirt6* promoter methylation in RA. **a**, localization of PVT1 in RA-FLSs detected by FISH (× 100); **b**, the CPG island of *sirt6* promoter region analyzed by bioinformatics website MethPrimer; **c**, the binding site between PVT1 and *sirt6* predicted by bioinformatics analysis; **d**, a bar chart of luciferase activity obtained from the dual-luciferase reporter gene assay; **e**, strips of *sirt6* promoter methylation in RA-FLSs of each group detected by MSP; **f**, the binding ability of *sirt6* promoter to DNA methyltransferase (DNMT1, DNMT3A and DNMT3B) in RA-FLSs measured by CHIP and RT-qPCR; **g**, the binding ability of PVT1 DNA methyltransferase (DNMT1, DNMT3A and DNMT3B) in RA-FLSs assayed by RIP and RT-qPCR; **h**, the mRNA expression of *sirt6* in RA-FLSs determined by RT-qPCR; **i**, the protein level of *sirt6* in RA-FLSs evaluated by western blot analysis; **j**, a bar chart of protein level of *sirt6* in RA-FLSs; ^#^ *p* < 0.05 vs. the sh-PVT1-NC group; ^&^ *p* < 0.05 vs. the oe-PVT1-NC group; ^*^ *p* < 0.05 vs. the blank group. The results of quantitative analysis were presented in mean ± standard derivation, which compared by one-way analysis of variance; the experimental data was obtained from the average of 3 independent experiments. PVT1, long non-coding RNA plasmacytoma variant translocation 1; Sirt6, sirtuin 6; RA-FLSs, rheumatoid arthritis fibroblast-like synoviocytes; GAPDH, glyceraldehyde-3-phosphate dehydrogenase; RT-qPCR, reverse transcription quantitative polymerase chain reaction; MSP, methylation specific PCR; FISH, fluorescence in situ hybridization; CHIP, chromatin immunoprecipitation; M, methylation; U, unmethylation; MUT, mutant; WT, wild type; DNMT1, DNA methyltransferase 1; DNMT3A, DNA methyltransferase 3A; DNMT3B, DNA methyltransferase 3B

In order to further elucidate the relationship between PVT1 and *sirt6* methylation, the localization of PVT1 in RA-FLSs was initially detected using FISH assay, the results (Fig. 7a) of which suggested that PVT1 was predominately expressed in the nucleus. Owing to the notion that PVT1 could potentially play a crucial role in the nucleus and participate in the regulation of gene transcription, we investigated whether PVT1 could bind to *sirt6* specifically using an online analysis software. The prediction results (Fig. 7b) revealed the existence of a specific binding region located between the *sirt6* promoter sequence and the PVT1 sequence. Furthermore, the enrichment status of CPG island in the *sirt6* promoter region was analyzed by bioinformatics analysis website MethPrimer (http://www.urogene.org/cgi-bin/methprimer/methprimer.cgi) (Fig. 7c), which demonstrated the existence of CPG island enrichment in the *sirt6* promoter region. In order to verify this prediction, the binding assay between PVT1 and *sirt6* promoter region was assessed using a dual-luciferase reporter gene assay. The results (Fig. 7d) of which indicated that the luciferase activity was decreased in the sirt6–5’UTR-WT + oe-PVT1 group when compared with that of the oe-PVT1-NC group (*p* < 0.05), however, while no significant difference was detected in relation to the luciferase activity in the sirt6–5’UTR-MUT + oe-PVT1 group (*p* > 0.05). The above results provided verification suggesting that PVT1 could specifically bind to *sirt6* promoter region.

Subsequently, 5′-Aza-d CdR group was set by incubating RA-FLSs with PBS dissolved DNA methyltransferase inhibitor 5-aza-2′-deoxycytidine, in the culture solution containing 50 μmol/L DMEM. The detection of methylation and unmethylation strips as well as the binding ability of PVT1 and sirt6 to DNA methyltransferase (DNMT1, DNMT3A and DNMT3B) in the blank, sh-PVT1-NC, sh-PVT1, oe-PVT1-NC, oe-PVT1, and 5′-Aza-d CdR groups were assessed by MSP, RT-qPCR, western blot analysis, CHIP and RIP assay. The methylation level of *sirt6* promoter detected by MSP (Fig. 7e) showed that both the methylated and unmethylated strips were located in *sirt6* promoter region of cells in the blank group; the unmethylated strip in *sirt6* promoter region of cells in the sh-PVT1 and 5′-Aza-d CdR groups; methylated strip in *sirt6* promoter region of cells in the oe-PVT1 group. The binding ability of PVT1 and *sirt6* promoter region to DNMT1, DNMT3A and DNMT3B was detected respectively by CHIP and RIP in combination with RT-qPCR, the results of which (Fig. 7f-g) showed a significantly decreased binding ability of PVT1 and *sirt6* promoter region to DNMT1, DNMT3A and DNMT3B in the sh-PVT1 and 5′-Aza-d CdR groups; while the oe-PVT1 group exhibited an enhanced binding ability of the PVT1 and *sirt6* promoter region to DNMT1, DNMT3A and DNMT3B. RT-qPCR and western blot analysis were used to determine the mRNA expression and protein levels of *sirt6* (Fig. 7h-j). The results illustrated that the mRNA expression and protein levels of *sirt6* were highly expressed in sh-PVT1 and 5′-Aza-d CdR groups, but poorly expressed in the oe-PVT1 group. Taken together, the results suggested that PVT1 inhibited the expression of *sirt6* by promoting the *sirt6* promoter methylation.

### PVT1 knockdown improves RA in rats

**Fig. 8:**
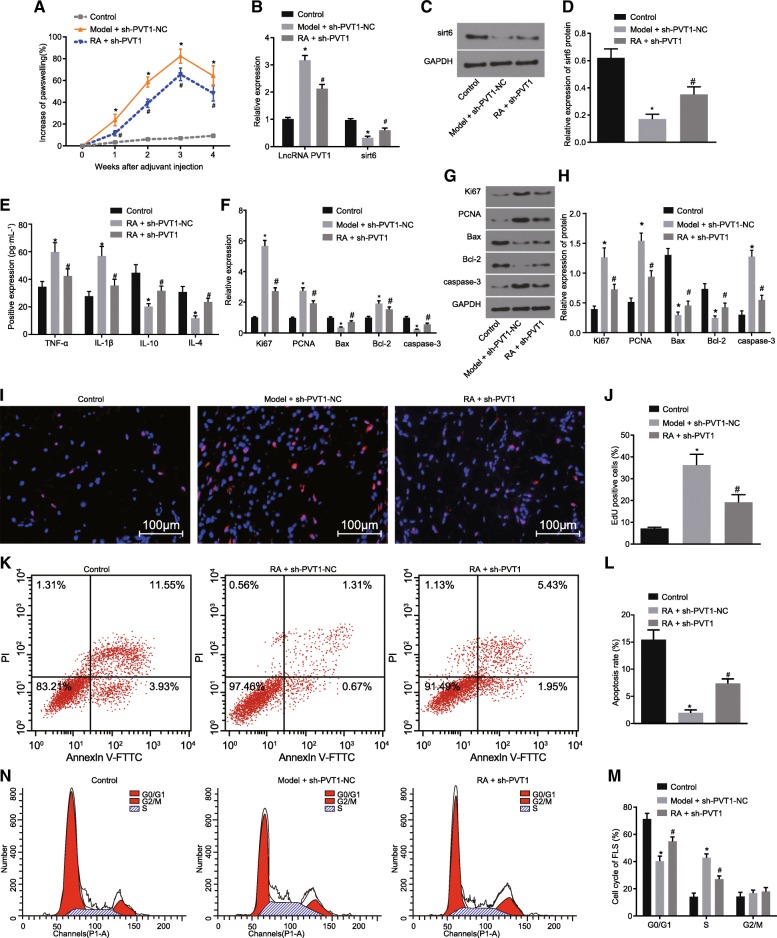
PVT1 knockdown helped to achieve RA amelioration via in vivo experiment. **a**, the percentage of increase of paw swelling of rats in each transfection group; **b**, PVT1 expression and mRNA expression of *sirt6* in RA-FLSs determined by RT-qPCR; **c**, protein level of *sirt6* in RA-FLSs determined by western blot analysis; **d**, the gray value analysis of *sirt6* in RA-FLSs; **e**, the serum levels of TNF-α, IL-1β, IL-10 and IL-4 measured by ELISA; **f**, the mRNA expression of Ki67, PCNA, Bcl-2, Bax and caspase-3 in RA-FLSs assessed by RT-qPCR; **g**, protein levels of Ki67, PCNA, Bcl-2, Bax and caspase-3 assessed by western blot analysis; **h**, protein levels of Ki67, PCNA, Bcl-2, Bax and caspase-3 in RA-FLSs; **i**, the RA-FLS proliferation determined by EDU staining (× 100); **j**, a bar chart of percentage of EDU positive stained RA-FLSs; **k**, the RA-FLS apoptosis evaluated by flow cytometry; **l**, a bar chart of apoptosis rate of RA-FLSs; **m**, the cell cycle distribution of RA-FLSs assessed by flow cytometry; **n**, a bar chart of percentage of cell cycle of RA-FLSs; ^*^ *p* < 0.05 vs. the control group; ^#^ *p* < 0.05 vs. the RA + sh-PVT1-NC group. The results of quantitative analysis were presented as mean ± standard derivation; one-way analysis of variance was performed for comparison; the experimental data was obtained from the average of 3 independent experiments. PVT1, long non-coding RNA plasmacytoma variant translocation 1; Sirt6, sirtuin 6; RA-FLSs, rheumatoid arthritis fibroblast-like synoviocytes; PCNA, proliferating cell nuclear antigen; Bcl-2, B-cell lymphoma/leukemia 2; Bax, Bcl-2-associated X protein; GAPDH, glyceraldehyde-3-phosphate dehydrogenase; TNF-α, tumor necrosis factor-α; IL-1β, interleukin-1β; IL-10, interleukin-10; IL-4, interleukin-4; EDU, 5-Ethynyl-2′-deoxyuridine; PI, propidium iodide; FITC, fluorescein isothiocyanate; ELISA, enzyme-linked immunosorbent assay; RT-qPCR, reverse transcription quantitative polymerase chain reaction; NC, negative control

In order to investigate the effects associated with PVT1 knockdown on the symptoms of RA-FLS, including inflammation, proliferation and apoptosis, the percentage of increase of paw swelling of rats in the control, RA + sh-PVT1-NC and RA + sh-PVT1 groups was calculated using a thickness measurement, with the expression levels of TNF-α, IL-1β, IL-10, IL-4, Ki67, PCNA, Bcl-2, Bax and caspase-3 determined by ELISA, RT-qPCR, western blot analysis, EDU staining and flow cytometry. Compared with the rats in the RA + sh-PVT1-NC group, rats in the RA + sh-PVT1 group exhibited a significantly decreased percentage of paw swelling (more serious than the control group) (Fig. 8a); decreased PVT1 expression, while elevated mRNA and protein levels of *sit6* (lower than the control group) (Fig. 8b-d); diminished serum levels of TNF-α and IL-1β (higher than the control group), while increased levels of IL-10 and IL-4 (lower than the control group) (Fig. 8e); decreased mRNA expression and protein levels of Ki67, PCNA and Bcl-2 (higher than the control group), while elevated levels of Bax and caspase-3 (lower than the control group) (Fig. 8f-h); suppressed cell proliferation (Fig. 8i-k); promoted cell apoptosis (Fig. 8l); increased percentage of cells at the G0/G1 phase, and decreased percentage of cells at the S phase (Fig. 8m and n) in RA-FLSs (all *p* < 0.05). Although signs of alleviated RA were identified in rats of the RA + sh-PVT1 group, the percentage of paw swelling was still higher than in rats of the control group. The rats in the RA + sh-PVT1 group still had a higher expression of TNF-α, IL-1β, Ki67, PCNA and Bcl-2, as well as a lower expression of IL-10, IL-4, Bax and caspase-3 than the rats in the control group. The results indicated that PVT1 knockdown contributed to RA alleviation in vivo.

## Discussion

RA-FLSs represent severe factors associated with joint cartilage erosion, as a consequence of RA-FLSs tumor-like behavior such as increased cell number, inflammatory response mediation, and aggressive phenotypes acquirement [17]. Accumulating literature has more recently placed an increasing emphasis on the important role of lncRNAs in the development and progression of RA including ZFAS1, whose upregulated expression has been implicated in RA-FLS with studies suggesting it contributes to the promotion of cell migration and invasion [18]. The long non-coding interleukin-7 receptor has been previously reported to promote RA-FLS proliferation, cell cycle distribution and hinder apoptosis [19]. During the current study, we aimed to investigate the functional roles of PVT1 in RA-FLS in an attempt to further elucidate the finer mechanisms associated with the pathogenesis of RA. Our key findings suggested that PVT1 knockdown suppressed RA-FLS proliferation, inflammation and facilitated apoptosis by upregulating *sirt6* via the inhibition of the *sirt6* promoter methylation.

A central finding of the current study revealed that the expression of PVT1 was elevated while the expression of *sirt6* was diminished in the synovial tissue as well as the RA-FLS of RA rats when compared with healthy controls. Besides, *sirt6* was verified to be regulated by PVT1 which could bind to *sirt6* promoter region. Upregulation of PVT1 in cancerous tissue has been flagged as an important player in the tumorigenesis of cancers including that of clear cell renal cell carcinoma [20], esophageal squamous cell carcinoma [21] and osteosarcoma [22], among which PVT1 knockdown or silencing was reported to exert a suppressive effect on the processes of proliferation, migration, and tumor growth. Studies have previously highlighted the potential of PVT1 as a therapeutic target for osteoarthritis with reports linking the upregulation of PVT1 and the distinct stimulation of chondrocyte apoptosis by sponging to microRNA-488-3p [13]. The mammalian sirtuin family is comprised of *sirt1* to *sirt7*, with *sirt6* reported to be located in the nucleus [23]. A previous study highlighted the potential anti-arthritic effects of *sirt6*, while the overexpression of *sirt6* has been demonstrated to alleviate the severity of arthritis, suppress the inflammatory response as well as improve the degree of joint destruction in collagen-induced arthritis mice [15]. The overexpression of *sirt6* has also been suggested to be a favorable factor in relation to chondrocyte replicative senescence prevention and IL-1β induced osteoarthritic changes [24]. Promoter hypermethylation is a crucial gene silencing mechanism that has been reported to be a potential indicator of future carcinogenesis [16]. PVT1 has been previously reported to play an oncogenic role in the process of prostate cancer with the downregulation of microRNA-146a expression by PVT1 promoting the methylation of the CPG island in its promoter region [25]. In the present study, methylation of sirt6 was identified in RA, as a result of the recruitment of DNA methyltransferases (DNMT1, DNMT3A and DNMT3B) to the *sirt6* promoter region by PVT1.

In an attempt to acquire a deeper understanding of the effects associated with PVT1 as well as *sirt6* on FLS proliferation and apoptosis, the expression of Ki67, PCNA, Bcl-2, Bax and caspase-3 in transfected cells was determined accordingly. The obtained results demonstrated that the expression of Ki67, PCNA and Bcl-2 was decreased while that of Bax and caspase-3 displayed elevated levels in RA-FLSs, in the event of PVT1 silenc or *sirt6* overexpression of PVT1. Upregulated PCNA has been identified in RA-FLSs, with its activation occurring as a result of enhanced cell migration and proliferation [26]. Moreover, PVT1 knockdown has been previously illustrated to result in decreased PCNA expression in gastric cancer cells which was found to inhibit cell proliferation [27]. Previously, the expression of Bcl-2, a well-documented anti-apoptotic gene, was found to be inhibited while that of Bax, a well-documented pro-apoptotic gene and the apoptotic protease caspase-3 was increased when the signal transducer and activator of transcription 3 was suppressed, ultimately inducing RA-FLS apoptosis [28]. Furthermore, CCRCC apoptosis induced by PVT1 knockdown has been previously achieved when the expression of poly ADP ribose polymerase and Bax protein was increased with the cell cycle arrested at the G1 phase [29]. Furthermore, the results of our study revealed that the knockdown of PVT1 or the overexpression of *sirt6* suppressed FLS inflammation while diminishing the serum levels of TNF-α and IL-1β and elevating the levels of IL-10 and IL-4 in the corresponding transfected RA rats. The pro-inflammatory cytokines TNF-α and IL-1β are produced by RA-FLS and inflammatory cells, both of which play a central role in the pathogenesis of RA [15]. As an anti-inflammatory cytokine, anti-inflammatory IL-10 plasmid DNA therapy has previously been employed to inhibit RA inflammation and joint destruction [30]. Furthermore, studies have shown that IL-4 promotes the expression of osteoprotegerin, with IL-4-knockout mice previously shown to exhibit extensive joint destruction, highlighting the anti-inflammatory and anti-osteoclastogenic role of IL-4 in RA [31]. In line with our study, previous evidence demonstrated up-regulation of *sirt6* suppressed inflammatory responses and bone destruction in mice with collagen-induced arthritis [15]. The analysis of our results combined with previous literature supported the notion that the knockdown of PVT1 plays a contributory role in the promotion of RA-FLS proliferation, inhibition of apoptosis and associated inflammation by upregulating *sirt6* through attenuating *sirt6* promoter methylation.

## Conclusion

**Fig. 9:**
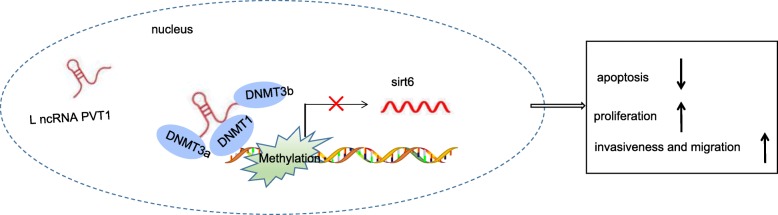
PVT1 could recruit DNA methyltransferases (DNMT1, DNMT3A and DNMT3B) to the *sirt6* promoter region, thereby inhibiting the transcription of *sirt6*. With the downregulated expression of *sirt6*, the RA-FLS apoptosis was promoted while proliferation and invasion were enhanced. PVT1, long non-coding RNA plasmacytoma variant translocation 1; Sirt6, sirtuin 6; RA-FLS, rheumatoid arthritis fibroblast-like synoviocyte; DNMT1, DNA methyltransferase 1; DNMT3A, DNA methyltransferase 3A; DNMT3B, DNA methyltransferase 3B

Taken together, the key findings of the current study present evidence suggesting that PVT1 regulates the proliferation, inflammation and apoptosis of RA-FLS in RA by regulating the expression of *sirt6* (Fig. 9). Our findings highlight the potential of PVT1 as a promising therapeutic target for RA treatment. The different effects of PVT1 on proliferation, inflammation and apoptosis in RA may be due to different downstream signal pathways, which would be interesting in our future research.

## Materials and methods

### Ethical statement

This study was performed in strict accordance with the guidelines in the Guide for the Care and Use of Laboratory Animals of the National Institutes of Health. Extensive efforts were made in order to minimize the suffering and number of animals used during the study.

### Establishment of RA model in rats

Twenty-five specific pathogen-free (SPF) female Lewis rats weighing between 100 and 197 g were obtained from the Beijing Vital River Laboratory Animal Technology Co., Ltd. (Beijing, China). After adaptively feeding for 1 week, 15 rats were randomly selected, anaesthetized with 3% pentobarbital sodium (P3761, Sigma-Aldrich, St. Louis, MO, USA) and treated with a single intradermal injection containing complete Freund’s adjuvant (CFA, heat-killed mycobacterium and clostridium butylicum were suspended in mineral oil, 10 mg/mL, 25 mg/kg) (Difico Lab., Detroit, MI, USA) into the tail base in order to establish a RA model. An additional 10 rats that were treated with a single intradermal injection with an equal volume of mineral oil used for the adjuvant preparation under diethyl ether anesthesia were used as control. Finally, the hind paw size (ankle thickness from the medial to the lateral malleolus) of the rats in each group was measured at day 0 after the administration of the adjuvant injection using a digital micrometer (Mitutoyo Corporation, Kanagawa Prefecture, Japan) to calculate the percentage of increase of paw swelling as compared with the 0th day [32].

### Construction of lentiviral vectors and screening

Based on the sequences of the PVT1 and *sirt6* transcripts in the GenBank database, sh-RNA and oe-RNA for target gene were designed. The specificity of the interference sequences as well as the control sequence were analyzed and subsequently confirmed by Homology Basic Local Alignment Search Tool (BLAST). Next, the designed sequences were synthesized by Shanghai Sangon Biotechnology Co., Ltd. (Shanghai, China) and used as a template for designing the polymerase chain reaction (PCR) primers. The forward primers were introduced with Xho I restriction sites and reverse primers with EcoR I restriction sites. Once the synthesized sh-RNA and oe-RNA had been verified by means of PCR, the double stranded interference sequence with cohesive ends was ligated with linearized p-LKO.1 lentiviral vector using a DNA ligation kit. Next, the ligated product was transformed into competent *Escherichia coli* cells, while the positive clones were selected for sequence verification purposes.

The 293 T cells at the logarithmic growth phase were seeded into 6-well plates with a density of 1 × 10^6^ cells/well and incubated overnight. When the cell confluence reached 80–90%, the recombinant interference plasmids with the correct sequence were co-transfected into the 293 T cells with package plasmid ps-PAX2 and envelope plasmid p-MD2.G respectively in accordance with the instructions of the Lipofectamine 2000 transfection reagent. After a 48 h period of transfection, the cells were centrifuged with the lentiviral particles-rich supernatant collected, which was then filtered through a 0.45 μm filter for cell debris removal and concentrated by centrifugation at 3221×g for 30 min using a 100 K pore-size ultrafiltration tube. Finally, the concentrated lentivirus was dispensed into eppendorf (EP) tubes and stored at − 80 °C for further experimentation.

### Rat grouping and lentiviral vector infection

Forty SPF female Lewis rats (weight: 100–191 g) purchased from Beijing Vital River Laboratory Animal Technology Co., Ltd. (Beijing, China) were placed on an adaptive diet 1 week, with 30 of the rats randomly selected for RA model establishment purposes. The rats selected were induced by CFA, and promptly injected with the lentiviral vectors following RA model establishment. Based on the injected vectors (treatments), the rats were divided accordingly into the RA + sh-PVT1-negative control (NC) (RA rats infected with sh-PVT1-NC lentiviral vectors into the tail base) and RA + sh-PVT1 (RA rats infected with sh-PVT1 lentiviral vectors into the tail base) groups. The remaining 10 rats were treated with a single intradermal injection comprised of an equal volume of mineral oil used for adjuvant preparation under diethyl ether anesthesia, which was used as the control.

### Isolation of RA-FLSs

The RA-FLSs of the knee joint were isolated from the RA rats under aseptic conditions, and detached with type I collagenase (1 mg/mL) for 3 h under 5% CO_2_. Then, RA-FLSs were subsequently filtered with a 100 μm pore-size cell filter, centrifuged, resuspended in Dulbecco’s Modified Eagle Medium (DMEM) supplemented with 10% fetal bovine serum (FBS) and cultured in a 5% CO_2_ incubator with saturated humidity at 37 °C. The medium was changed every other day, with the poorly adhered cells removed by means of phosphate buffer saline (PBS) washing. The cells were passaged 3–4 times, until the cell confluence was noted to be greater than 80%, after which the cells were treated with 2.5 g/L trypsin and passaged. Cell morphology was finally analyzed under an inverted microscope.

### Characterization of RA-FLSs

The cells in all the passages were collected, detached and resuspended into cell suspension (1 × 10^9^ cells/L). The cells were then subjected to indirect immunofluorescence staining using rabbit anti-mouse CD55 (RA-FLS surface marker) mAb (ab232688, Abcam Inc., Cambridge, MA, USA). The mouse monoclonal antibody to Immunoglobulin M (IgM) (ab91545, Abcam Inc., Cambridge, MA, USA) was regarded as the isotype NC. After that, CD55 was detected by means of flow cytometry and analyzed by Cell-Quest software. The Vimentin expression of the RA-FLSs was detected by means of Immunohistochemical staining based on the provided instructions of the Immunohistochemistry kit. The coverslips at the 4th passage were fixed with acetone, blocked with H_2_O_2_, and treated in a routine manner with normal non-immune serum, mouse anti-human vimentin antibody (1: 100), biotin-labeled anti-mouse antibody, streptomycin avidin-peroxidase solution and diaminobenzidine (DAB) solution in a sequential fashion. Finally, the expression of Vimentin in RA-FLSs was detected. The isolated and identified RA-FLSs were then further cultured for subsequent experiments.

### Cell grouping and transfection

The RA-FLSs were adherently cultured in conditions of 5% CO_2_ and 37 °C in high glucose DMEM containing 10% FBS. The RA-FLSs at the logarithmic growth phase were collected and seeded into 6-well plates with a density of 1 × 10^6^ cells/well. After cells were cultured for 24 h, the original medium in wells was discarded, with 1 mL of culture medium containing hexamethylene bromide (a final concentration of 8 μg/mL) and 100 μL corresponding lentiviral vectors subsequently added to the wells. The cells were then grouped into the (1) blank (cells not transfected with the any lentiviral vectors), (2) sh-PVT1-NC (cells transfected with the sh-PVTl-NC lentiviral vectors), (3) sh-PVT1 (cells transfected with the sh-PVT1 lentiviral vectors), oe-PVT1-NC (cells transfected oe-PVT1-NC lentiviral vectors), (4) oe-PVT1 (cells transfected oe-PVT1 lentiviral vectors), (5) oe-sirt6-NC (cells transfected oe-sirt6-NC lentiviral vectors), oe-sirt6 (cells transfected with oe-sirt6 lentiviral vectors), (6) sh-sirt6-NC (transfected sh-sirt6-NC lentiviral vectors), sh-sirt6 (cells transfected sh-sirt6 lentiviral vectors), (7) oe-PVT1-NC + oe-sirt6-NC (cells co-transfected with oe-PVT1-NC and oe-sirt6-NC lentiviral vectors) (8) oe-PVT1 + oe-sirt6 (cells transfected with oe-PVT1 and (8) oe-sirt6 lentiviral vectors, and (9) oe-PVT1 + oe-sirt6-NC (cells transfected with oe-PVT1 and oe-sirt6-NC lentiviral vectors). Due to the framework of p-LKO.1 lentiviral vector containing the puromycin resistance gene, the infected cells were able to be screened by inoculating the cells with a medium containing puromycin (a final concentration of 3 μg/mL) 12 h post lentiviral vector infection, with the puromycin medium changed every day. When the cells reached 80–90% confluence, they were transferred to culture flasks and continually inoculated in puromycin medium for subsequent experiments.

### Immunofluorescence assay

Four weeks after RA rat model establishment, the rats were euthanized with the injection of 30 mg/kg pentobarbital sodium. After the synovial tissues had been collected, they were fixed with 4% paraformaldehyde, conventionally dehydrated, cleared, immersed in wax, embedded and sectioned (4-μm thickness) for immunofluorescence detection purposes. The tissue sections were then dewaxed in water, treated with 0.3% H_2_O_2_ for 5–10 min at room temperature in order to inactivate the endogenous enzyme, followed by three PBS washes (5 min/time). The sections were subsequently immersed in 0.01 mol/L citrate buffer solution (pH 6.0) and heated until boiling point (repeated 1–2 times after 5–10 min interval). The sections were then subjected to antigen retrieval by heating, and then cooled at room temperature. Next, normal goat serum blocking reagent was added for section blockade purposes at room temperature for 20 min, followed by incubation with primary rabbit anti-mouse antibody to *sirt6* mAb (ab191385, 1: 1000, Abcam Inc., Cambridge, MA, USA) at 4 °C for 24 h and with the secondary antibody Alexa Fluor® 647-labeled goat anti-rabbit Immunoglobulin G (IgG) (ab150077, 1: 1000, Abcam Inc., Cambridge, MA, USA) at 37 °C for 30 min. Finally, the nucleus was counterstained with 4′,6-diamidino-2-phenylindole (DAPI), rinsed, dried, and mounted with 90% glycerol. The images were obtained and analyzed under a fluorescence microscope, with the positive area of immunofluorescence in each field analyzed using Image J software to calculate the mean fluorescence intensity.

### Reverse transcription quantitative polymerase chain reaction (RT-qPCR)

The total RNA in the synovial tissue was extracted in accordance with the Trizol one-step method based on the instructions of the Trizol kit (Invitrogen Inc., Carlsbad, CA, USA). The fluorescence quantitative PCR was conducted according to the instructions of the SYBR® Premix Ex TaqTM II Kit. After successful RT, the target gene as well as the internal reference gene were amplified using a fluorescence quantitative PCR instrument (ABI7500, Applied Biosystems, Carlsbad, CA, USA) with glyceraldehyde-3-phosphate dehydrogenase (GAPDH) regarded as the internal reference. The primer sequences are depicted in Table 1. PVT1 expression as well as the mRNA expression of *sirt6*, Ki67, proliferating cell nuclear antigen (PCNA), B-cell lymphoma/leukemia 2 (Bcl-2), Bcl-2-associated X protein (Bax), and caspase-3 was calculated by 2^-ΔΔCt^ method [33]. Finally, the total RNA in RA-FLSs was extracted from each group after a 48 h period of incubation, with the mRNA expression determined by RT-qPCR methods after transfection [34].

**Table 1:** RT-qPCR primer sequences

Gene	Sequences (5′ - 3′)
LncRNA PVT1	F: TGGTACCGAGCTCGGATCCTCAAGATGGCTGTGCC
	R: CCGCCACTGTGCTGGATGATAGAAAAAGAATTTAATAG
*sirt6*	F: GCCGTCTGGTCATTGTCA
	R: AGCCTTGGGTGCTACTGG
Ki67	F: ACCTACCTTCAACGCTCTCTGA
	R: TCCGCTTACTTCTGGACAATCT
PCNA	F: ACCGCTGCGATCGCAACCTG
	R: GGCATGTTTACTACGCAGCTG
Bax	F: ATGCGTCCACCAAGAAGC
	R: CAGTTGAAGTTGCCATCAGC
Bcl-2	F: GACTGAGTACCTGAACCGGCATC
	R: CTGAGCAGCGTCTTCAGAGACA
caspase-3	F: AAGCGTGCCAACGACAGCTGTGGGAC
	R: TTAATAGTCATCATATTGACACTGGC
GAPDH	F: TTGGTATCGTGGAAGGACTCA
	R: TGTCATCATATTTGGCAGGTT

Note: RT-qPCR, reverse transcription quantitative polymerase chain reaction; LncRNA PVT1, long non-coding RNA plasmacytoma variant translocation 1; Sirt6, sirtuin 6; PCNA, proliferating cell nuclear antigen; Bcl-2, B-cell lymphoma/leukemia 2; Bax, Bcl-2-associated X protein; casp-3, caspase-3; GAPDH, glyceraldehyde-3-phosphate dehydrogenase; F, forward; R, reverse

### Western blot analysis

The synovial tissue that had been preserved in liquid nitrogen was subjected to total protein extraction. A total of 15 μL of protein underwent electrophoresis using 5% concentration gel and a 15% spacer gel, and the proteins on the separation gel was transferred onto a membrane. Membrane blockade was then performed using Tris-buffered saline with Tween 20 (TBST) containing 5% bovine serum albumin (BSA) for 1 h. After the blocking solution had been removed, the membrane was placed in plastic grooves and incubated with 5% BSA diluted primary antibodies rabbit anti-mouse *sirt6* (ab191385, 1: 2000), Ki67 (ab16667, 1: 1000), PCNA (ab18197, 1: 1000), Bax (ab32503, 1: 1000), Bcl-2 (ab196495, 1: 1000), and caspase-3 (ab13847, 1: 500) in a 4 °C refrigerator overnight. The next day, the membrane was incubated with diluted secondary antibody goat anti-rabbit IgG (ab6721, 1: 2000) at 4 °C for 4–6 h. All the aforementioned antibodies were purchased from Abcam Inc. (Cambridge, MA, USA). The chemiluminescence reagents were subsequently added to the membrane in an even manner whereby the images were developed with a developing solution. All western blotting bands were subjected to relative optical density (OD) analysis [35]. Finally, the total RNA in RA-FLSs at the logarithmic growth phase was extracted from each group after transfection, with the western blot analysis methods employed to determine the protein level after transfection using the aforementioned protocol.

### Enzyme-linked immunosorbent assay (ELISA)

Four weeks after RA rat model establishment, the rats were euthanized by means of injection with 30 mg/kg pentobarbital sodium with 2 mL of blood collected from the plexus venosus ophthalmicus to collect the supernatant for ELISA analysis by centrifugation at 3221×g for 15 min. Next, post-transfection RA-FLSs at the logarithmic growth phase were resuspended and seed in 24-well plates (1 × 10^6^ cells/well). After a 24 h period of culture, the supernatant of the cell culture medium was collected for centrifugation purposes at 4 °C at 1812×g for 1 min, from which, the supernatant was used to detect the serum levels of the inflammatory cytokine tumor necrosis factor-α (TNF-α), interleukin-1β (IL-1β), interleukin-10 (IL-10) and interleukin-4 (IL-4) according to the instructions of ELISA kit TNF-α (ab208348, 1: 1000), IL-1β (ab100704, 1: 1000), IL-10 (ab108870, 1: 1000), and IL-4 (ab221833, 1: 1000). All the ELISA kits were purchased from Abcam Inc. (Cambridge, MA, USA).

### Fluorescence in situ hybridization (FISH)

FISH assay was employed in order to identify the subcellular localization of PVT1 in RA-FLSs. According to the instructions of Ribo™ lncRNA FISH Probe Mix (Red) (RiboBio Co., Ltd., Guangzhou, Guangdong, China), the coverslips were placed in the 6-well plates, with the cells seeded into the plates for 1 day until they had reached 80% confluence. Next, the coverslips were removed, fixed in 1 mL 4% paraformaldehyde at room temperature after PBS washing and then treated with glycine and phalide. The cells were subsequently added with 250 μL prehybridization solution and incubated at 42 °C for 1 h, followed by incubation with 250 μL hybridization solution containing 300 ng/mL PVT1 probe at 42 °C overnight after the prehybridization solution had been removed. On the next day, the cells were washed three times with PBS, and stained with diluted DAPI staining solution (DAPI: PBST = 1: 800) in a 24-well plate for 5 min for nucleus staining purposes. After an additional 3 PBS washes (3 min/time), the coverslips were mounted with anti-fluorescence quencher followed by fluorescence analysis. Five different fields were selected under the guidance of a fluorescence microscope (Olympus, Tokyo, Japan), with images subsequently acquired.

### Dual-luciferase reporter gene assay

The target genes of PVT1 were predicted in connection with the bioinformatics prediction website, with the dual-luciferase reporter gene assay employed to verify the prediction. The promoter region of the target gene, in this case, *sirt6*, was cloned and amplified. The plasmid was extracted based on the instructions of the Omega Plasmid Mini Kit (D6943–01, Beijing Think-Far Technology Co., Ltd., Beijing, China), and then subjected to double restriction enzyme digestion using restriction endonuclease Xba I and Xho I. The target fragment was ligated with dual-luciferase reporter vector pGL3-basic and the ligated product was transformed into *Escherichia coli* DH5H cells for plasmid amplification. The wild type dual-luciferase reporter vector was referred to as WT, which was employed as a reference in the construction of the mutant vector named MUT using the site-directed mutagenesis method. The recombinant plasmids were termed pGL3-basic-sirt6–5’untranslated region (UTR)-WT and pGL3-basic-sirt6–5’UTR-MUT, respectively. The sequence-verified WT and MUT plasmids were respectively co-transfected with oe-PVT1-NC and oe-PVT1 into the adherent HEK-293 T cells (K1223, Invitrogen, Carlsbad, California, USA) that were seeded in 6-well plates (2 × 10^5^ cells/well) prior to transfection. The successfully transfected cells were continued to culture for 48 h, and the cells were collected to detect the luciferase activity of PVT1 on sirt6–5’UTR according to the instructions of Dual Luciferase Assay Kit (ELDL-100, Bioassay Systems, Hayward, CA, USA) using a fluorescence detector (Glomax 20/20, Promega, Madison, WI, USA).

### Methylation specific PCR (MSP)

The RA-FLSs after transfection was lysed with proteinase K, with the lysate subjected to DNA extraction using an animal genomic DNA extraction kit. The extracted DNA was further treated with hydrosulfite, purified with Wizard resin (Promega, Madison, WI, USA), treated with NaOH, precipitated with ethanol, and dissolved in water. The PCR primers (Table 2) were designed based on the methods reported by Herman et al. The sirt6-methylated (M) and sirt6-unmethylated (U) specific primers were purchased from Invitrogen Inc. (Carlsbad, CA, USA). Finally, after the PCR products had been electrophoresed on 1.5% agarose gel (Sangon Biotechnology Co., Ltd., Shanghai, China), and stained with Gelred (Nucleic acid dyes, Biotium, Hayward, CA, USA), and the products were visualized using the Polar Ultraviolet Imager (Bio-Rad Inc., Hercules, CA, USA).

**Table 2:** MSP primer sequences

Gene	Sequences (5′ - 3′)
sirt6-methylation	F: GTGTAACGGAGTAAGGGATGTTAC
	R: ACTAAACCCATATAAATTCACCGAA
sirt6-unmethylation	F: TGTGTAATGGAGTAAGGGATGTTAT
	R: ACTAAACCCATATAAATTCACCAAA

Note: MSP, methylation specific polymerase chain reaction; Sirt6, sirtuin 6; F, forward; R, reverse

### Chromatin immunoprecipitation (CHIP) assay

The CHIP kit (Millipore Corp, Billerica, MA, USA) was employed to investigate the enrichment of DNA methyltransferase 1 (DNMT1) in *sirt6* gene promoter region. When the RA-FLSs reached 70–80% confluence, the cells were fixed with 1% formaldehyde at room temperature for 10 min for DNA and protein cross-linking in the cells by means of immobilization. After cross-linking, the cross-linked DNA/protein product was randomly fractured into a suitable size by ultrasonic treatment (each for 10s, 10 s interval with a total of 15 cycles). The fragments were centrifuged (4 °C) at 30237×g for supernatant collection. A total of 3 tubes of supernatant were collected with each of one incubated at 4 °C overnight with the positive control antibody (RNA polymerase II), NC antibody (normal mouse IgG) and target protein-specific antibodies [mouse anti-DNMT1 (ab13537, 1: 100), rabbit anti-DNMT3A (ab2850, 1: 100) and rabbit anti-DNMT3B (ab2851, 1: 100)], respectively. All the antibodies were purchased from Abcam Inc. (Cambridge, MA, USA). The endogenous DNA-protein complex was precipitated using Protein Agarose/Sepharose, with the supernatant discarded after a brief period of centrifugation. The non-specific complex was washed, de-crosslinked at 65 °C overnight, and purified by phenol/chloroform for DNA fragment retrieval purposes. Finally, the enrichment of DNMT1 in *sirt6* gene promoter region was assessed by RT-qPCR using sirt6-methylation specific primers (Table 2).

### RNA binding protein immunoprecipitation (RIP) assay

RIP was processed based on the instructions of Magna RIP RNA-Binding Protein Immunoprecipitation kit (Millipore Corp, Billerica, MA, USA). RA-FLSs were collected and washed twice with pre-cooled PBS, and then incubated with 100 μL lysate buffer containing protease inhibitor and ribonuclease inhibitor on ice for 30 min before centrifugation (4 °C) at 28985×g for 3 min. A small amount of the supernatant was obtained and used as the Input positive control, with the rabbit anti-mouse IgG (ab7099, 1: 100) regarded as the NC. The remaining supernatant was incubated with 10–50 μL protein A/G-beads together with1 μg antibodies of mouse anti-DNMT1 (ab13537, 1: 100), rabbit anti-DNMT3A (ab2850, 1: 100) and DNMT3B (ab2851, 1: 100) antibodies, respectively, in a rotating incubator at 4 °C for overnight. The aforementioned antibodies above were purchased from Abcam Inc. (Cambridge, MA, USA). On the next day, the mixture was centrifuged (4 °C) at 1812×g for 5 min to remove the supernatant with 1 mL of protein A/G-beads pellet washed with lysate buffer 3–4 times, followed by centrifugation (4 °C) at 201×g for 1 min after each wash. The washed pellet was heated with 15 μL 2 × sodium dodecyl sulfate loading buffer in boiling water for 10 min for RNA isolation using the Trizol one-step method. The interaction between DNMT1 and PVT1 was verified by RT-qPCR analysis using PVT1-specific primers with GAPDH employed as the internal control.

### 5-Ethynyl-2′-deoxyuridine (EDU) staining

The RA-FLSs in each group were cultured in DMEM containing 10% FBS and double antibodies (penicillin/streptomycin), as well as the RA-FLSs at the logarithmic growth phase collected, resuspened, and divided into 96-well plates at a density of 5 × 10^3^ cells/well, followed by the addition of 100 μL of culture medium containing EDU solution (50 μmol/L) for incubation. After that, 50 μL glycine (2 g/L) was added in each well for 5-min incubation. An additional 100 μL of PBS containing 0.5% TritonX-100 was added in each well for 10-min incubation. The cells were subsequently stained with 100 μL 1 × Apollo reaction solution for 30 min at room temperature under conditions void of light. Following the removal of staining solution, 100 μL PBS containing 0.5% TritonX-100 was added in each well, followed by the addition of 100 μL methanol for 1–2 washes (5 min/time) after the penetration solution had been removed. A total of 100 μL 1 × Hoechst 33342 reaction solution was added in each well for 30-min incubation at room temperature without light. Finally, the cells were analyzed under a fluorescence microscope, with images photographed at a 550 nm excitation channel and a 350 nm excitation channel in which the red stained cells were reflective of the proliferated cells and the blue stained cells representative of the total cells. A total of 3 visual fields were randomly selected under a fluorescence microscope at a magnification of 400 × in order to count the EDU stained cells (proliferated cells) and Hoechst 33342 stained cells (the total cells) for calculation of the EDU stained positive cell percentage using this formula: EDU positive rate (%) = (the number of EDU positive nucleus / the number of total nucleus) × 100% [36]. The RA-FLSs at the logarithmic growth phase post transfection were collected for cell proliferation evaluation using the aforementioned EDU staining.

### Flow cytometry

The RA-FLSs in each transfection group were cultured in DMEM containing 10% FBS and penicillin and streptomycin. The cells were treated with ethylene diamine tetraacetic acid (EDTA)-free trypsin, and collected into EP tubes. After two PBS washes, the cells were fixed in paraformaldehyde for 1 h, centrifuged at 201×g for 5 min and resuspended in precooled ethanol. The cells were then incubated with 500 μL propidium iodide (PI)-RNase staining solution for 15 min at room temperature under conditions void of light after ethanol removal and two additional PBS washes. Before cell cycle observation using a flow cytometer (FACSCantoTM II, Becton, Dickinson and Company, Franklin Lakes, NJ, USA), cells were filtered into EP tubes through filter membranes.

Apart from PI staining, the cells were also resuspended in 100 μL 1 × Binding Buffer for Annexin-V-fluorescein isothiocyanate (FITC) staining. Cell suspension was incubated with 5 μL FITC and 5 μL PI at room temperature for 30 min under dark conditions, followed by resuspension in 400 μL 1 × Binding Buffer and filtration into a flow tube through filter membrane. Finally, cell apoptosis was detected using the flow cytometer.

### Statistical analysis

SPSS 21.0 statistical software (IBM Corp. Armonk, NY, USA) was employed for data analysis purposes. Measurement data were presented as mean ± standard deviation. The normal distribution and variance homogeneity were also examined. In the event that the data followed normal distribution and had even variance, then an independent *t*-test was applied for comparison between the two groups. The comparison between the multiple groups analyzed by one-way analysis of variance or repeated measures analysis of variance. In addition, intra-group comparison was performed using a post-hoc test. All tests were bilateral. A *p* value less than 0.05, was considered to be statistically significant.

## Data Availability

The datasets generated during the current study are available.

## Abbreviations

BLAST: Basic Local Alignment Search Tool
CHIP: Chromatin immunoprecipitation
EDU: 5-Ethynyl-2′-deoxyuridine
ELISA: Enzyme-linked immunosorbent assay
FBS: Fetal bovine serum
FISH: Fluorescence in situ hybridization
GAPDH: Glyceraldehyde-3-phosphate dehydrogenase
lncRNAs: Long non-coding RNAs
MSP: Methylation specific PCR
PCNA: Proliferating cell nuclear antigen
PCR: Polymerase chain reaction
PI: Propidium iodide
PVT1: Plasmacytoma variant translocation 1
RA: Rheumatoid arthritis
RA-FLS: RA fibroblast-like synoviocyte
SFs: Synovial fibroblasts
